# Mini-biography for Mr. Xitao Cai: the pioneer botanist of the plant kingdom

**DOI:** 10.1007/s13238-018-0564-1

**Published:** 2018-07-06

**Authors:** Gaibian Wang, Quanxing Zhang, Yongping Yang

**Affiliations:** Kunming Institute of Botany, Chinese Academy of Sciences, Kunming, 650201 China

**Figure 1:**
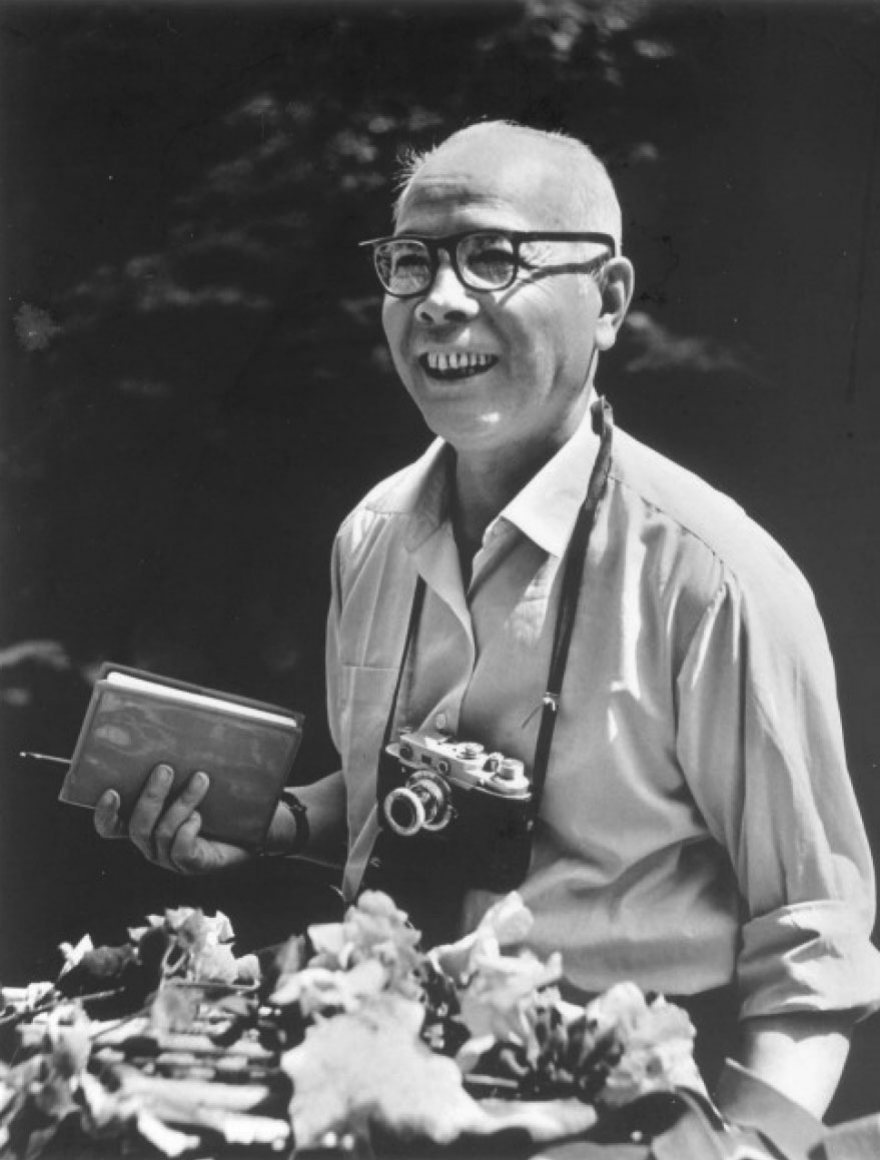
Mr. Xitao Cai (Hse-Tao TSAI, April 10th, 1911–March 9th, 1981)

On March 9th, 1981, Mr. Xitao Cai (Hse-Tao Tsai) (Fig. 1) one of the most famous pioneer botanists in China, passed away at his age of 70 in Kunming. It has been 37 years since he left us. Mr. Cai founded Yunnan Institute of Agricultural and Forestry Botany in 1938, the predecessor of Kunming Institute of Botany, Chinese Academy of Sciences. At the celebration of the 80th anniversary for the institute, we deeply cherish Mr. Cai, the founder of the institute, who has dedicated his entire life to the development of botany science in China.

**Figure 2:**
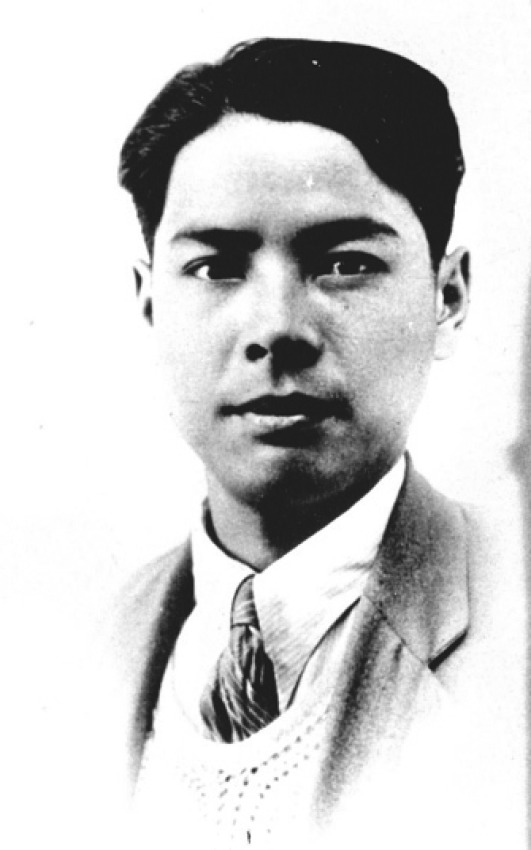
Mr. Xitao Cai (1933)

**Figure 3:**
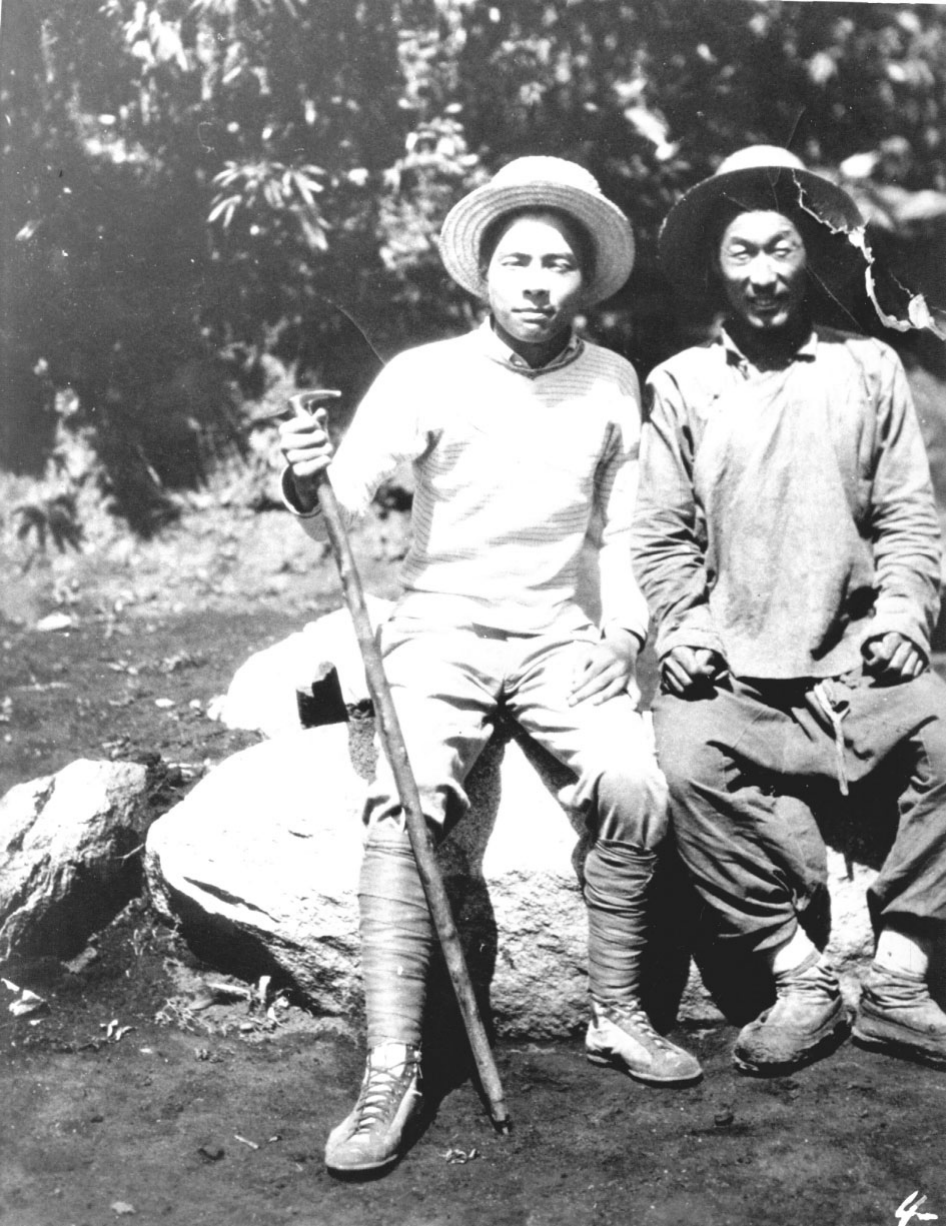
Mr. Xitao Cai (left) on the field trip to Nujiang Prefecture of Yunnan (1932)

Mr. Xitao Cai was born in Zhejiang Province on April 10th, 1911. He was admitted into the Department of Physics in Kwang Hua University in Shanghai in 1929. During his studies in Shanghai, he frequently visited his brother-in-law, Mr. Wangdao Chen and was influenced by many revolutionary predecessors and literary giants, such as Mr. Qiubai Qu, Mr. Da Li, Mr. Zhengnong Xia, Mr. Lu Xun, Mr. Xuefeng Feng and Mr. Yuzhi Hu. In September 1930, recommended by Mr. Wangdao Chen, Mr. Cai became a trainee in the Fan Memorial Institute of Biology. During this time, Professor Xiansu Hu (Hsen-Hsu Hu), the Director of the institute, often sent him to collect plant specimens near Peking. The next year, Mr. Cai (Fig. 2) published a research paper *An Study on Lamiaceae in Sichuan Province* co-author with Prof. Hu (Xun et al., 1993). In February 1932, Mr. Cai was dispatched to Yunnan on a botanical expedition organized by the Fan Memorial Institute of Biology (Fig. 3). He traveled through Yunnan, risked his life and conducted field survey and plant collection in the remote mountainous areas. During this expedition, he collected more than 21,000 specimens including 427 new species, which unveiled the mask of Yunnan Province as a “Plant Kingdom” and contributed significantly to the development of the plant science in Yunnan. Later on, Mr. Cai translated the book *Origin of Cultivated Plants* written by de Candolle into Chinese, together with Mr. Dejun Yu (Te-Tsun Yü) (Jiang, 2017), and published a series of research papers on the family Leguminosaceae, Rosaceae and the genus *Amorphophallus* plants. His early works enabled the Chinese botanical community to have better understandings about the origins of crops and served as invaluable references for the following studies till now. In addition, Mr. Cai was inspired by the customs of the ethnic minorities and wrote several essays which has been described as “very impressive” by famous Chinese writer, Mr. Lu Xun.

In 1937, the War of Resistance against Japan broke out. Many institutions in Peking started to move to Southwest China. The Fan Memorial Institute of Biology planned to establish a botanical research base in Yunnan. Mr. Cai moved to Yunnan with his family. In July 1938, Yunnan Institute of Agricultural and Forestry Botany, the predecessor of Kunming Institute of Botany, was established in the Heilongtan Park, Kunming City. Mr. Cai was appointed as the park manager as well. In the spring of 1940, the institute bought a land site near the Heilongtan Park to construct the office building. And the construction of the office building was completed in 1941. The motto “Explore every mountain and river, name every grass and tree” was carved in stone and embedded into the wall. Mr. Zhengyi Wu (Cheng-Yhi Wu), who once took students from the Southwest Associated University for internships in Yunnan Institute of Agricultural and Forestry Botany, described the institute as “the highest botanical institution in China at that time” and “a research center for plant taxonomy in old China” in his commemorative essay to Mr. Cai (Wu, 1991).

In 1945, after the victory of the Resistance against Japanese Aggression War, many institutions moved back from Yunnan Province. With the outbreak of the civil war, prices soared and it became very difficult to live. In order to maintain the life of the employees and protect more than 100,000 plant specimens, Mr. Cai organized the employees to grow vegetables, flowers and tobacco to save themselves. He opened a parrot shop to sell cutting flowers, ornamental plants and pets, and struggled till the liberation of Kunming. For the institute, Mr. Cai was not only the founder and the actual manager, but also the one who persisted from the beginning to the end. In the essay of *Recalling Mr. Xitao*’*s life* by Prof. Fenghuai Chen (Feng-Hwai Chen), who worked together with Mr. Cai during 1930s–1940s. It is said that, “Mr. Cai made the most important contributions to the establishment of Yunnan Institute of Agricultural and Forestry Botany.”

**Figure 4:**
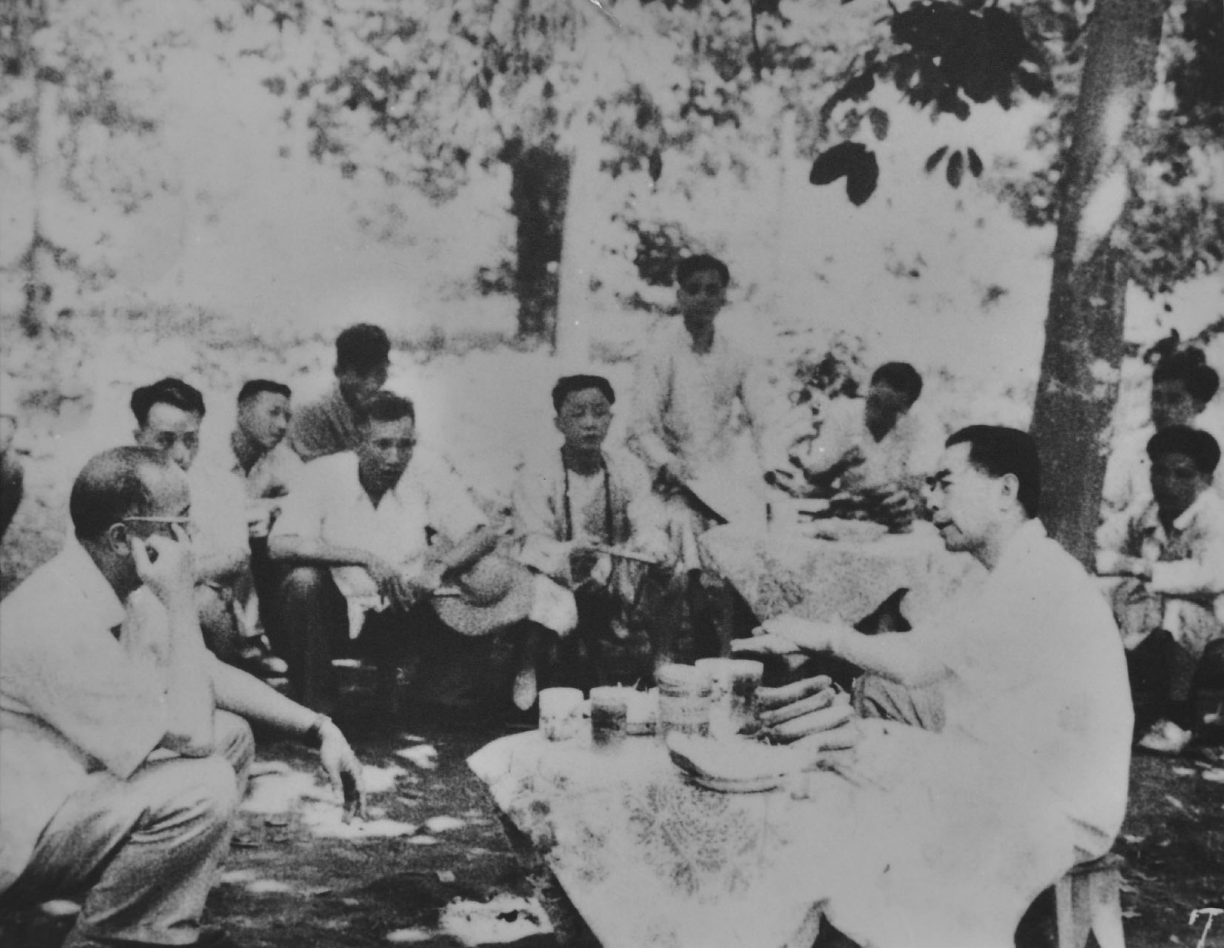
Premier Zhou Enlai had a cordial talk with Mr. Cai (right) under rubber forest in Xishuangbanna

In April 1950, Yunnan Institute of Agricultural and Forestry Botany was merged and re-named as Kunming Station of the Institute of Plant Taxonomy, Chinese Academy of Sciences. Mr. Cai was appointed as the director of Kunming Station. With the special care from Premier Zhou Enlai (Fig. 4), Mr. Cai focused on the development and construction. The campus of Kunming Station was expanded, three modern buildings used as administration office and research laboratories were constructed. In research fields, Mr. Cai established the first chemistry laboratory to study medicinal and aromatic plants (the predecessor of the State Key Laboratory of Phytochemistry and Sustainable Use of Plant Resources of Kunming Institute of Botany). Later on, Kunming Station was upgraded as an independent institute, Kunming Institute of Botany, Chinese Academy of Sciences. Mr. Zhengyi Wu was appointed as the Director and Mr. Xitao Cai as the deputy director.

In 1959, under the propose and leadership of Mr. Cai, Kunming Institute of Botany started to found the first tropical botanical garden of China in Huludao Peninsula, Xishuangbanna, South Yunnan. Mr. Cai made great contribution to the foundation of botanical garden, and all institute staff were deeply touched by Mr. Cai’s scientific spirit and noble personalities. Mr. Cai and his team have overcome many unimaginable difficulties and made great achievements in the construction of the garden and scientific researches. They also cultivated many talents who later became famous scientists in China and abroad. In 1978, the tropical botanical garden was renamed as Yunnan Institute of Tropical Botany, the predecessor of Xishuangbanna Tropical Botanical Garden, Chinese Academy of Sciences (Qin, 1991).

As early as 1940s, Mr. Cai paid attention to tobacco production in Yunnan. In 1945, his team succeed in the domestication and cultivation of Mammoth Gold in Kunming, a tobacco cultivar introduced by Mr. Huanyong Chen (Woon-Young Chun) (Huang, 2016) from Virginia, USA. Mr. Cai organized a series of training workshops for tobacco cultivar selection, demonstration and large-scale cultivation, and made earliest contributions for tobacco production in Yunnan. In the 1950s, he led an investigation team and explored the rubber resources in Yunnan. He proposed that Xishuangbanna was the most suitable place for rubber tree plantation, which was adopted by the State Council to build the rubber plantation farm in China. And as a member of national rubber research team, Mr. Cai was awarded the First Prize for National Invention by the National Science Committee in 1982. Furthermore, he and his team explored and introduced many important medicinal plants, oil plants, aromatic and spice plants, valuable fast-growing species and so on, such as dragon blood trees, hodgsonia squash, camphor oil tree and mytenus plants. They have provided new resources and technologies for the economic and social development in the tropical areas in China.

As the saying goes, “it takes ten years to grow a tree but it takes a hundred years to rear people”. As a master in science, Mr. Cai guided many projects and researches, established lots of scientific research divisions and promoted a great deal of scientific research findings. As an educator, Mr. Cai has brought up numerous professionals and therefore built up a multi-disciplinary research team. He taught the younger generations English, Latin, botanical courses and guided them in botanical expedition, plant domestication, phytochemical analysis, plant cultivation and scientific paper writing. He clearly stated that the young scientists should learn in practice, accumulate and expand their knowledge and explore their own way to success. Mr. Yaozong Feng, one of Mr Cai’s students, wrote in *Teacher Cai Xitao—My Guide in Scientific Exploration*, “Mr. Cai is not only my enlightenment teacher but also the leader that guides me along all the time”.

Mr. Cai has made outstanding contributions to botanical researches in Yunnan, the “Plant Kingdom”. His devotion to science will always be an inspiration for people in Xishuangbanna Tropical Botanical Garden and Kunming Institute of Botany, two botanical institutions founded by him.
